# An energy-efficient in-memory computing architecture for survival data analysis based on resistive switching memories

**DOI:** 10.3389/fnins.2022.932270

**Published:** 2022-08-09

**Authors:** Andrea Baroni, Artem Glukhov, Eduardo Pérez, Christian Wenger, Enrico Calore, Sebastiano Fabio Schifano, Piero Olivo, Daniele Ielmini, Cristian Zambelli

**Affiliations:** ^1^IHP-Leibniz Institut fur Innovative Mikroelektronik, Frankfurt (Oder), Germany; ^2^Dipartimento di Elettronica, Informazione e Bioingegneria, Politecnico di Milano and IU.NET, Milano, Italy; ^3^BTU Cottbus-Senftenberg, Cottbus, Germany; ^4^Dipartimento di Fisica e Scienze Della Terra, Università Degli Studi di Ferrara, Ferrara, Italy; ^5^Istituto Nazionale di Fisica Nucleare (INFN), Ferrara, Italy; ^6^Dipartimento di Scienze Dell'Ambiente e Della Prevenzione, Università Degli Studi di Ferrara, Ferrara, Italy; ^7^Dipartimento di Ingegneria, Università Degli Studi di Ferrara, Ferrara, Italy

**Keywords:** resistive RAM (RRAM), drift, in-memory computing (IMC), survival analysis, multi level conductance

## Abstract

One of the objectives fostered in medical science is the so-called precision medicine, which requires the analysis of a large amount of survival data from patients to deeply understand treatment options. Tools like machine learning (ML) and deep neural networks are becoming a de-facto standard. Nowadays, computing facilities based on the Von Neumann architecture are devoted to these tasks, yet rapidly hitting a bottleneck in performance and energy efficiency. The in-memory computing (IMC) architecture emerged as a revolutionary approach to overcome that issue. In this work, we propose an IMC architecture based on resistive switching memory (RRAM) crossbar arrays to provide a convenient primitive for matrix-vector multiplication in a single computational step. This opens massive performance improvement in the acceleration of a neural network that is frequently used in survival analysis of biomedical records, namely the DeepSurv. We explored how the synaptic weights mapping strategy and the programming algorithms developed to counter RRAM non-idealities expose a performance/energy trade-off. Finally, we discussed how this application is tailored for the IMC architecture rather than being executed on commodity systems.

## 1. Introduction

In the last decade, medical researchers have started to extensively rely on machine learning (ML) and artificial neural networks (ANNs) to gain further insights into large amounts of complex and intertwined data (Anaya-Isaza et al., 2021; Allegra et al., 2022). Records concerning patients' clinical and genetic features, pathologies, interventions, hospitalizations, and follow ups are deeply investigated through survival analysis models, whose goal is to provide *ad hoc* treatment options and ultimately shed light on the origins of the disease (Wu et al., 2018). State-of-the-art data analysis platforms are built on Von Neumann computing architectures that devise bulky and power-hungry central processing units (CPUs), graphic processing units (GPUs), and memory devices embedded in high performance computing (HPC) machines (Bajaj and Ansari, 2021). The frequent data movement caused by the performance mismatch between the computing elements and the memory units in these machines is rapidly hitting the so-called “Von Neumann bottleneck” (Zou et al., 2021). Speed and energy efficiency in data analysis are therefore in jeopardy. One of the main candidates to overcome this issue materialized in revolutionary computing architecture, namely the in-memory computing (IMC) concept. The IMC bases on high density crossbar arrays constituted by memory devices that offer high throughput, low energy, and good scaling features (Zahoor et al., 2020; Mannocci et al., 2022). Among the many memory technologies proposed in the last years for IMC integration resistive-switching, non-volatile memory (RRAM) has been identified as an ideal candidate (Burr et al., 2015; Zidan et al., 2018; Mannocci et al., 2022). RRAM enables massive IMC parallelism in performing the matrix-vector-multiplication (MVM) in one computational step (i.e., one clock cycle) *via* the physical laws of Ohm and Kirchhoff in the analog domain (Hu et al., 2018; Ielmini and Wong, 2018; Ma et al., 2018; Yu, 2018). However, despite these promising properties, RRAM devices have physical limitations that may threaten the MVM execution. This would result in failures of the ML and ANN tasks based on that operation. The limited tunability of the conductance levels in RRAM devices is one of the most tedious issues exposed in IMC accelerators based on this technology. Non-idealities like the device-to-device (D2D) and the cycle-to-cycle (C2C) variability (Fantini et al., 2013; Ambrogio et al., 2014a; Grossi et al., 2016), the random telegraph noise (RTN) (Ambrogio et al., 2014b, 2015a; Chai et al., 2018; Du et al., 2020), the random walk (Ambrogio et al., 2015b), and the conductance drift (Kang et al., 2017; Lin et al., 2019; Baroni et al., 2021) impair the multi-level conductance (MLC) capability of the RRAM technology resulting in lower bit precision with respect to CPUs and GPUs. Those limitations can be overcome through the application of tailored programs and verified algorithms that accurately set the RRAM in the desired conductance state (Pérez et al., 2019). However, the stochastic nature of the technology hardly questions its effectiveness. In Kang et al. (2017), Yu et al. (2020), and Milo et al. (2021), it has been demonstrated that when those techniques are applied there is a significant drift of the conductance distributions both in short and long time scales. Such behavior is an additional non-ideality that limits the accuracy of the MVM and should be countered with a drift-safe algorithm (Baroni et al., 2021) that are in turn less energy-efficient. This calls for a design space exploration of IMC architectures devoted to specific ML and ANN tasks execution.

In this work, we present an IMC architecture based on RRAM technology that implements a deep neural network for survival analysis of biomedical data, namely the DeepSurv (Katzman et al., 2018). The design of the network back-annotates the physical characteristics of 4 kbits RRAM arrays. We study how different MLC programming algorithms impact the performance and the energy consumption of the neural network especially when the drift phenomenon takes place. Furthermore, we analyze how different synaptic weights quantization strategies can expose a performance/energy trade-off. Finally, we demonstrate that an RRAM-based design of the DeepSurv is better executed on an IMC concept rather than a commodity GPU-accelerated Von Neumann architecture both in terms of throughput and energy efficiency.

## 2. Materials and methods

### 2.1. Survival analysis through deep neural networks

Survival data are commonly used in medical research to develop models that assess the significance of prognostics variables in outcomes such as patient's death or disease recurrence (Bair and Tibshirani, 2004; Royston and Altman, 2013). The survival analysis requires a patient's baseline data *x* (i.e., the variables), an event time *T*, and an indicator variable *E* built on the presence/absence of a specific event (e.g., death, disease relapse, etc.). Survival probability *S*(*t*) and hazard rate λ(*t*) can be then computed. The former is indicated as *S*(*t*) = *P*(*T*>*t*), which represents the probability that a patient has survived beyond a time *t*, while the latter is calculated as


(1) $$\begin{array}{r} \lambda \left(t\right)=\underset{\epsilon \to 0}{\lim}\frac{P\left(t\leq T<t+\epsilon |T\geq t\right)}{\epsilon } \end{array}$$

which defines the probability that a patient will not survive an extra infinitesimal amount of time ϵ, given its survival up to time *t*.

**Figure 1:**
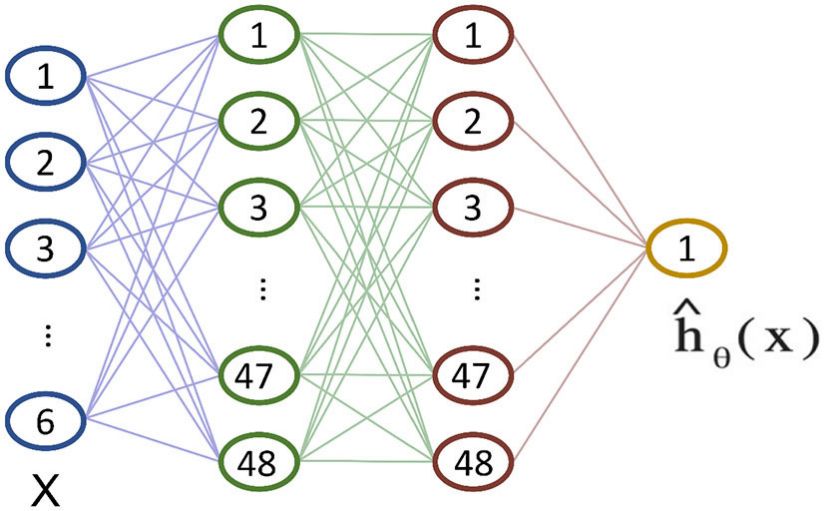
The structure of the DeepSurv neural network according to the implementation provided in Katzman et al. (2018). The dropout layers between inputs and hidden layers are not shown for clarity.

The Cox Proportional Hazards (CPH) model is a common semi-parametric survival analysis framework that relates a patient's survival given their baseline data *x* (Therneau and Grambsch, 2000). The model assumes that the hazard function is composed of two non-negative functions: a baseline hazard function λ_0_(*t*) and a risk score *r*(*x*) = *e*^*h*(*x*)^. Following the notations in Therneau and Grambsch (2000), the CPH estimates the function *h*(*x*) through a linear function, so that ${ĥ}_{\beta }\left(x\right)=\beta^{T}x$. The parameters β in the model are fine-tuned to properly model the hazard rate function. However, in many medical scenarios (Bice et al., 2020; Byun et al., 2021; Hadanny et al., 2022), the assumption of a linear log-risk function (i.e., *h*(*x*)) may be too simplistic. To this extent, Katzman et al. (2018) developed the DeepSurv feed-forward neural network whose non-linear output ĥ_θ_(*x*) replaces the linear combination of features ĥ_β_(*x*). DeepSurv is a configurable neural network whose structure is depicted in Figure 1. It consists of several fully-connected layers followed by dropout layers. The final layer of the network is a single neuron that performs a linear combination of the hidden features and outputs the risk function ĥ_θ_ (*x*).

In this work, we applied the DeepSurv to the Worcester Heart Attack Study (WHAS) dataset (Hosmer et al., 2008) consisting of a total of 1,638 observations and 5 patients' features: age, sex, body-mass-index (BMI), left heart failure complications (CHF), and order of Myocardial Infarction (MIORD). A total of 42.1% percent of patients died during the study with a median death time of 516 days. We fixed the neural network hyper-parameters as suggested in Katzman et al. (2018) to enable a fair comparison between its GPU implementation and the proposed RRAM. To summarize, the network consists of an input layer with 6 neurons (5 patient features + 1 bias) and two dense hidden layers with 48 neurons each. All the layers are followed by a dropout layer featuring a ReLU activation function and the output layer features a linear activation.

To assess DeepSurv's predictive accuracy on the WHAS dataset, we measure Harrell's concordance-index (C-index) (Harrell et al., 1984). Its goal is to reflect how well the neural network predicts the patients' death times. A C-index = 1 represents a perfect prediction.

### 2.2. RRAM technology and algorithms

**Figure 2:**
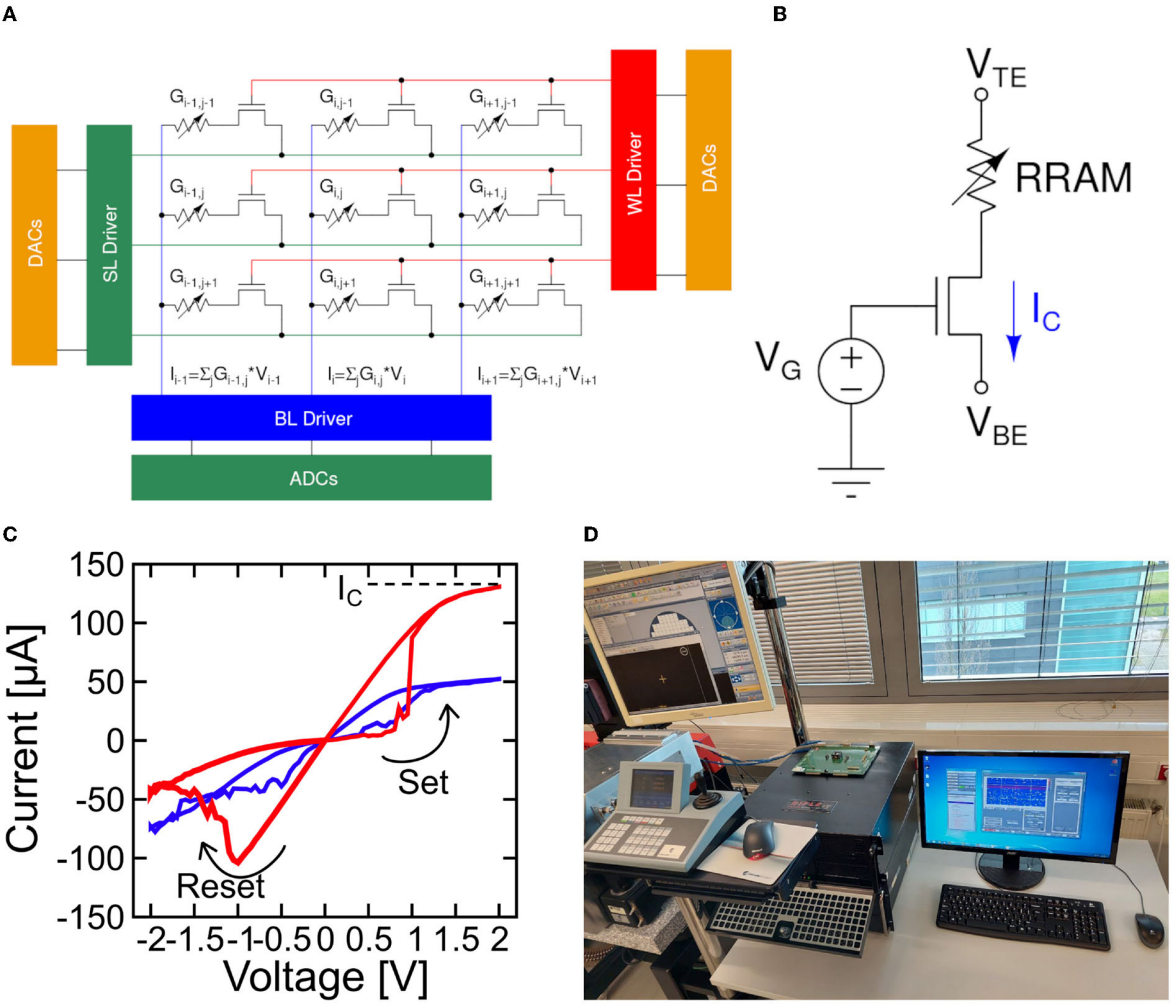
**(A)** Crossbar array architecture for matrix-vector-multiplication (MVM) operations. **(B)** Schematic of a 1T1R resistive switching memory (RRAM) device integrated into the 4 kbits array used in this work. **(C)** I–V characteristics of a 1T1R RRAM device measured for increasing *V*_*G*_ proving multi-level conductance (MLC) capability. **(D)** The RIFLE test equipment used in this work.

Figure 2A illustrates the implementation of an MVM operation in a generic DeepSurv's layer by using a crossbar architecture. Additional circuitry like the Analog-to-Digital converter (ADC), the Digital-to-Analog converter (DAC), and wordline/bitline/sourceline drivers are evidenced. The single elements of the crossbar are constituted by 1T1R RRAM devices, whose structure is depicted in Figure 2B. The memristive element consists of a materials stack made by a 150 nm TiN top and bottom electrodes (TE and BE, respectively) deposited by magnetron sputtering, a 7 nm Ti layer (acting as oxygen scavenging layer under the top electrode) and an 8 nm HfO_2_ switching layer grown by atomic layer deposition (ALD) (Grossi et al., 2018). The transistor in series to the memristive cell is a 0.25 μm nMOS from IHP Microelectronics. Its 2-fold role is to select a cell in the crossbar and to provide a proper compliance current (*I*_*C*_) for switching operations *via* the gate voltage *V*_*G*_. Figure 2C shows the current-voltage (I-V) characteristics of an RRAM device, exhibiting a tunable MLC operation that is sought for MVM operation. The measurements of this work are performed on 4 kbits RRAM crossbar arrays featuring 64 wordlines and 64 bitlines. A microphotograph of the chip is available in Zambelli et al. (2015). All the experiments were performed on quad flat packaged (QFP) devices with the RIFLE Automated Test Equipment from ActiveTechnologies (see Figure 2D).

**Figure 3:**
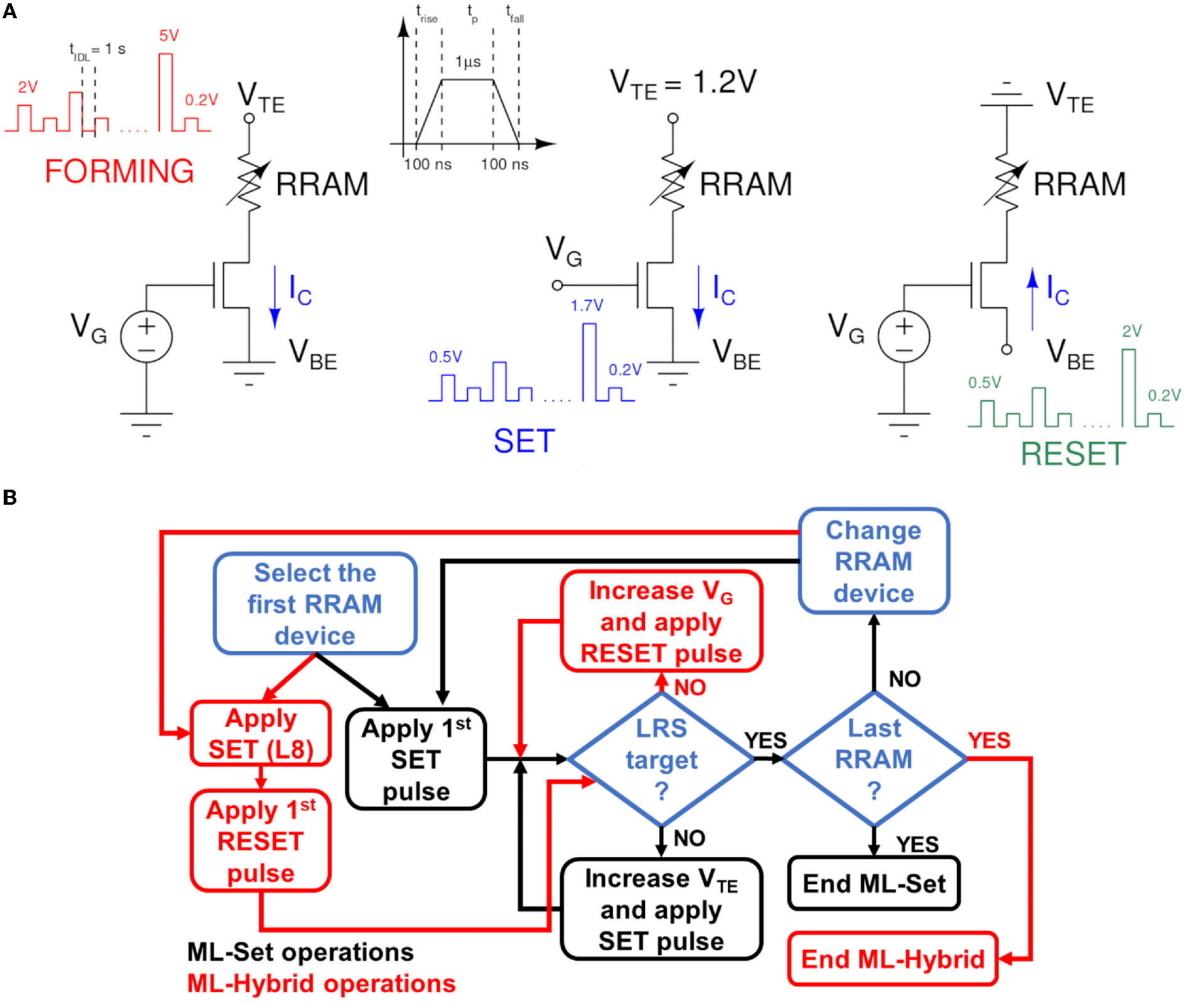
**(A)** Algorithms used in RRAM forming, set, and reset operation modes. The voltages during the operation and the verify phases are evidenced. **(B)** Global flowchart scheme, representing the different steps for ML-SET and ML-Hybrid algorithms.

The RRAM devices in the array are prepared for conductance switching through a forming operation using the Incremental Step Pulse program and Verify Algorithm (ISPVA) proposed in Pérez et al. (2019). The gate voltage *V*_*G*_ is set to 1.4 V and the top electrode voltage *V*_*TE*_ is gradually increased from 2 to 5 V in steps of 10 mV. The target conductance for the operation has been chosen as 200 μS to avoid RRAM cells premature wear-out. After the Forming, we performed a reset operation to bring all the cells to the lowest conductance state, namely L1 at 25 μS. The reset uses the ISPVA in which the bottom electrode voltage *V*_*BE*_ is swept from 0.5 to 2 V with 100 mV steps. The *V*_*G*_ is set to 2.7 V to ensure a high *I*_*C*_ is required to disrupt the conductive filament in the RRAM cell. Different approaches to achieve multiple conductance states have been demonstrated for this technology. The approach in Milo et al. (2021) modulates the *I*_*C*_ of the set operation through a program and verify algorithm. With such methodology, eight linearly spaced conductance levels (L2-L9) between 50 and 225 μS are obtained plus L1. These values are the target conductances *G*_*trg*_ checked during the verify operation (i.e., equivalent to a read). In the set operation for multilevel conductance tuning, the *V*_*G*_ is gradually incremented from 0.5 to 1.7 V in 10 mV steps, featuring 1 μs pulse duration (*t*_*p*_). The delay between the pulse and the consecutive verify is about 1 s. Both the rise *t*_*rise*_ and the fall time *t*_*fall*_ of the pulses is set to 100 ns. The *V*_*TE*_ is chosen to be 1.2 V. We refer to this algorithm as the ML-Set. Its characteristics are depicted in Figure 3 along with the ones in forming and reset operations.

A second approach proposed in the literature is that of Baroni et al. (2021). In this case, instead of starting from the L1 distribution and applying an ML-Set algorithm to reach the higher conductance state, we start from the L8 distribution and reach L2-L5 through a controlled reset operation. The L6-L9 distributions are still obtained with the ML-Set. We named this approach as ML-Hybrid algorithm since it embodies two different switching operations of the RRAM cells in the array. To avoid the over-stress of the device, we performed the incremental gate voltage and verify algorithm (IGVVA) reset experiments with a *V*_*BE*_ set to 1.2 V and sweeping *V*_*G*_ from 1.5 to 2.9 V in steps of 10 mV. Figure 3 shows the summarized programming operation used in the two different algorithms and a flowchart that follows their procedures step by step.

**Figure 4:**
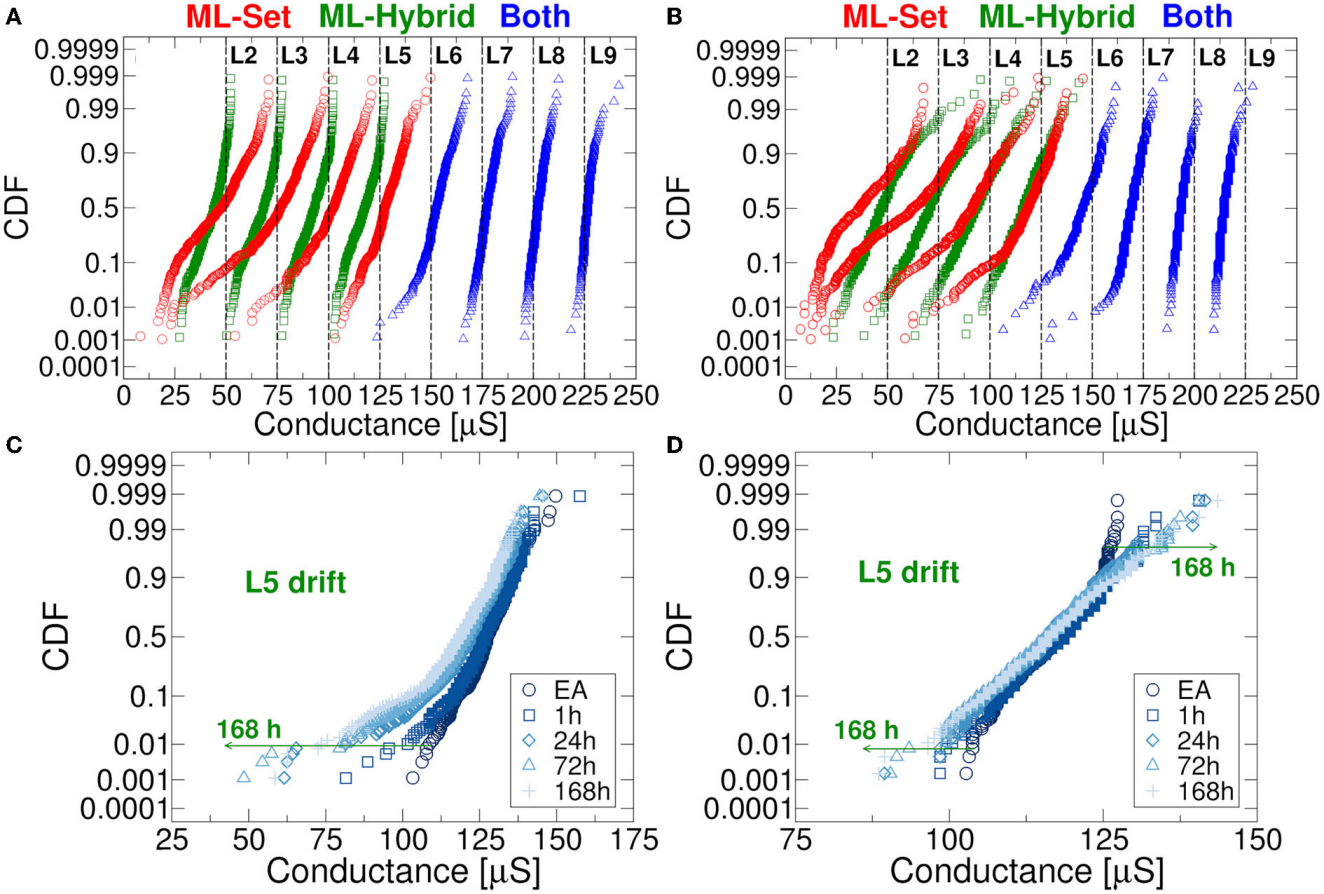
**(A)** L2-L9 levels distribution in RRAM obtained after the application of the programming algorithm. **(B)** L2-L9 levels distribution in RRAM after 168 h evidencing the drift. **(C)** Evolution in time of the L5 distribution for ML-Set algorithm. **(D)** Same study performed for the ML-Hybrid algorithm.

Figure 4A compares the conductance distributions obtained with ML-Set and ML-Hybrid algorithms considering 1024 RRAM cells per conductance level. It can be noted that in the former there is a significant drift of the distributions exhibiting cells whose conductance falls below their desired *G*_*trg*_. Such drift happens in the very first minutes after the application of the programming algorithm. It is easy to observe that the L2-L5 conductance levels are the most affected by the drift and that this phenomenon can lead to larger instabilities over time if not properly handled (Baroni et al., 2021). Instead, in the latter algorithm, the drift seems mitigated for L2-L5 levels, but a small fraction of cells has an inverted trend in terms of conductance shift. The total number of displaced cells far from *G*_*trg*_ is however lower although at the price of a larger power consumption paid during the programming of the RRAM cells (a reset followed by a set operation is needed). We must also remind that the conductance drift is a process that lasts also for longer times after the end of the programming algorithms. Figure 4B displays the behavior of the L2-L9 levels after 168 h for both programming approaches. The choice of the L8 conductive level as the starting point for ML-Hybrid is ascribed to reliability considerations. This conductive level is the same *G*_*trg*_ used for the Forming operation. We have observed that starting the ML-Hybrid operations from L9 can introduce a high number of stuck-at-L9 cells, therefore reducing the population of available cells for the study. To this extent, we chose L8 to reduce possible reliability threats that could hamper the interpretations of this study. Figures 4C,D show, without lack of generality, the evolution of the drift of the L5 distribution in 168 h after the application of the programming algorithms. Once again, the ML-Hybrid seems to perform better in terms of drift countering. These peculiar technology characteristics are now considered for the RRAM-based DeepSurv implementation. The choice of 168 h was made after experimental observations accrued in our previous work (Baroni et al., 2021). In the experimental section of that work, we could see that between 100 and 168 h (1 week), the drift phenomenon tends to reach a saturation point, no longer showing a progressive and additional degradation of the RRAM device conductances at room temperature. We, therefore, considered the situation at 168 h at room temperature as the worst case for our analysis.

### 2.3. Weights quantization for RRAM-based DeepSurv implementation

The RRAM crossbar implementation of the DeepSurv requires the synaptic weights mapping as finite conductance values in single 1T1R devices. Due to the physical nature of the RRAM technology investigated in this work, we experienced that a single device can be programmed only with 9 discrete conductance levels (see Figure 4), thus representing only positive weights. However, in state-of-the-art neural networks, both positive and negative values are required with a numerical precision in the order of 32 or even 64 bits. To overcome such limitations, we can represent each weight *W* using a differential approach. To this extent, two separate conductance values mapped in two 1T1R devices, namely *G*^+^ and *G*^−^, are devised. By subtracting them in the analog domain, we obtain the desired value *W* = *G*^+^−*G*^−^, as described in Milo et al. (2021). This approach allows mapping the negative weights of the network as well, while inherently increasing the number of levels that can be mapped on RRAM devices from 9 to 17. Despite such improvement, we are still far from the radix used by CPUs or GPUs arithmetic units (i.e., 32/64 bits).

**Figure 5:**
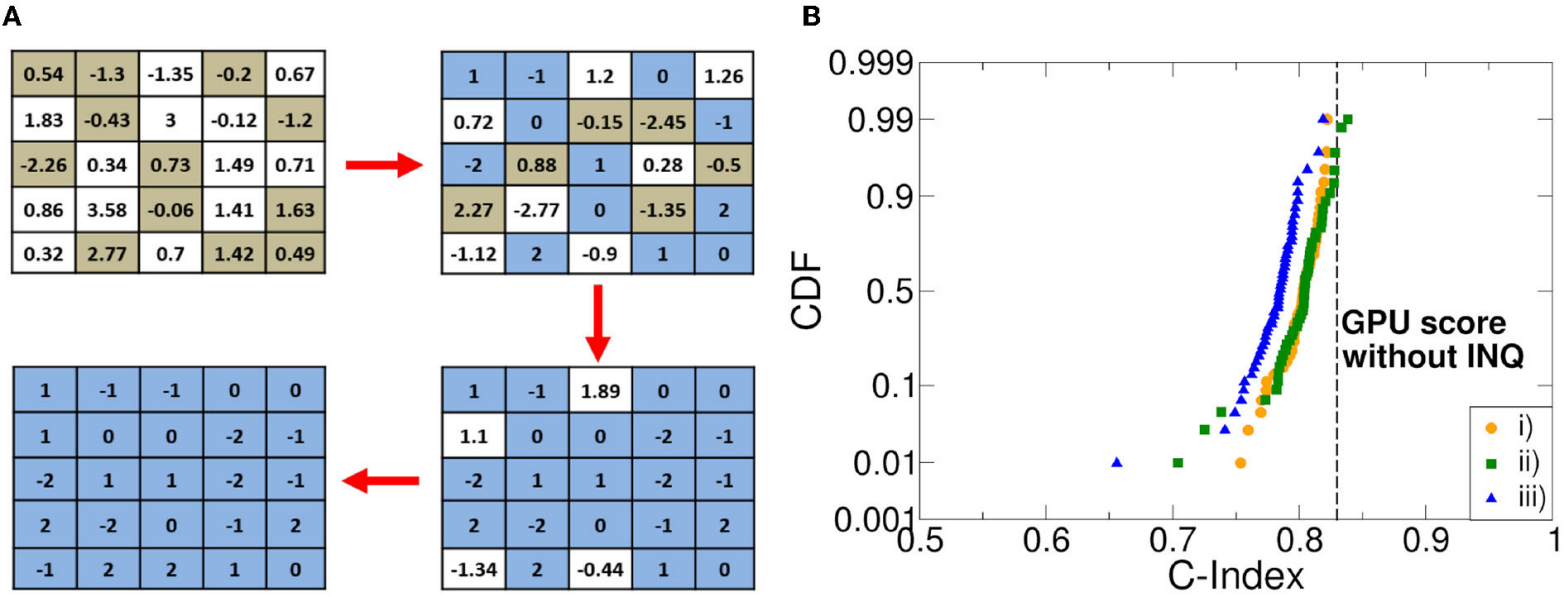
**(A)** An example of the application of the incremental network quantization (INQ). algorithm. **(B)** cumulative distribution function (CDF) of the C-index retrieved in Monte Carlo simulations with different weight picking strategies compared with the C-index obtained by a graphic processing unit (GPU) without quantization in training and working with full floating-point precision.

The numerical precision of the synaptic weights can be reduced without compromising the network accuracy through a quantization algorithm. As a preliminary step, we trained the DeepSurv network on a K80 Nvidia GPU with full floating-point precision using Tensorflow 2 (Keras 2.3) for Python 3.8 compiled with CUDA 11.4 and cuDNN 8.2 support. The training time was 17.32 s using 500 epochs. Then, we implemented an iterative training algorithm described in Zhou et al. (2017) as an incremental network quantization (INQ). The key is to build an RRAM-aware training operation through the decision of the quantization steps number that is to be followed. Straightforwardly, the larger the number of steps the longer the training of the network will take. On the other hand, if the number of steps proves to be too small, the network could become unstable or drastically degrades its accuracy. During our experiments, we found an acceptable trade-off in using 4 incremental quantization steps: 50, 75, 87, and 100%. These percentage values allow deriving the number of weights in each DeepSurv's layer that will be rounded to the nearest quantization level at the end of each training epoch of the network while leaving the remaining weights free to continue with the training non-quantized. The advantage of this strategy lies in the compensation of the quantization-induced non-idealities (Zhou et al., 2017). Figure 5A depicts an example of the INQ procedure application.

Once we found the right compromise between DeepSurv's accuracy and stability, we had to choose the quantization strategy. Indeed, it is mandatory to define a policy related to the weights choice during INQ, so that they can be quantized in a precise order. In this work, three different priority patterns were studied: *i)* weights with the greatest absolute value; *ii)* weights with the lowest absolute value; and *iii)* weights featuring the lowest quantization error. The quantization error refers to the value calculated as the absolute difference between the value of the weight at the end of the training and the value of the closest quantization level. Figure 5B reports the cumulative distribution function (CDF) of the obtained C-index in 100 training experiments. The C-index obtained by training a GPU without INQ strategies and with a full floating-point precision is also reported for sake of comparison. We experienced that while the policy *i)* leads to optimal results, it is also the one leading to a higher network instability materialized in the impossibility to complete the training in some cases. Policy *ii)* has similar behavior with respect to the former one, although a slightly larger variability is experienced in favor of higher training stability. Policy *iii)* achieves a slightly lower C-index mean and a larger variability with respect to the other policies. We were able to observe that the best C-index values were reached when the weights with the lowest quantization error coincided with those with the lowest absolute value. Conversely, we get worse C-index values when the weights predominantly coincide with those selected in *i)*. For these reasons, we performed all our experiments with policy *ii)*.

**Figure 6:**
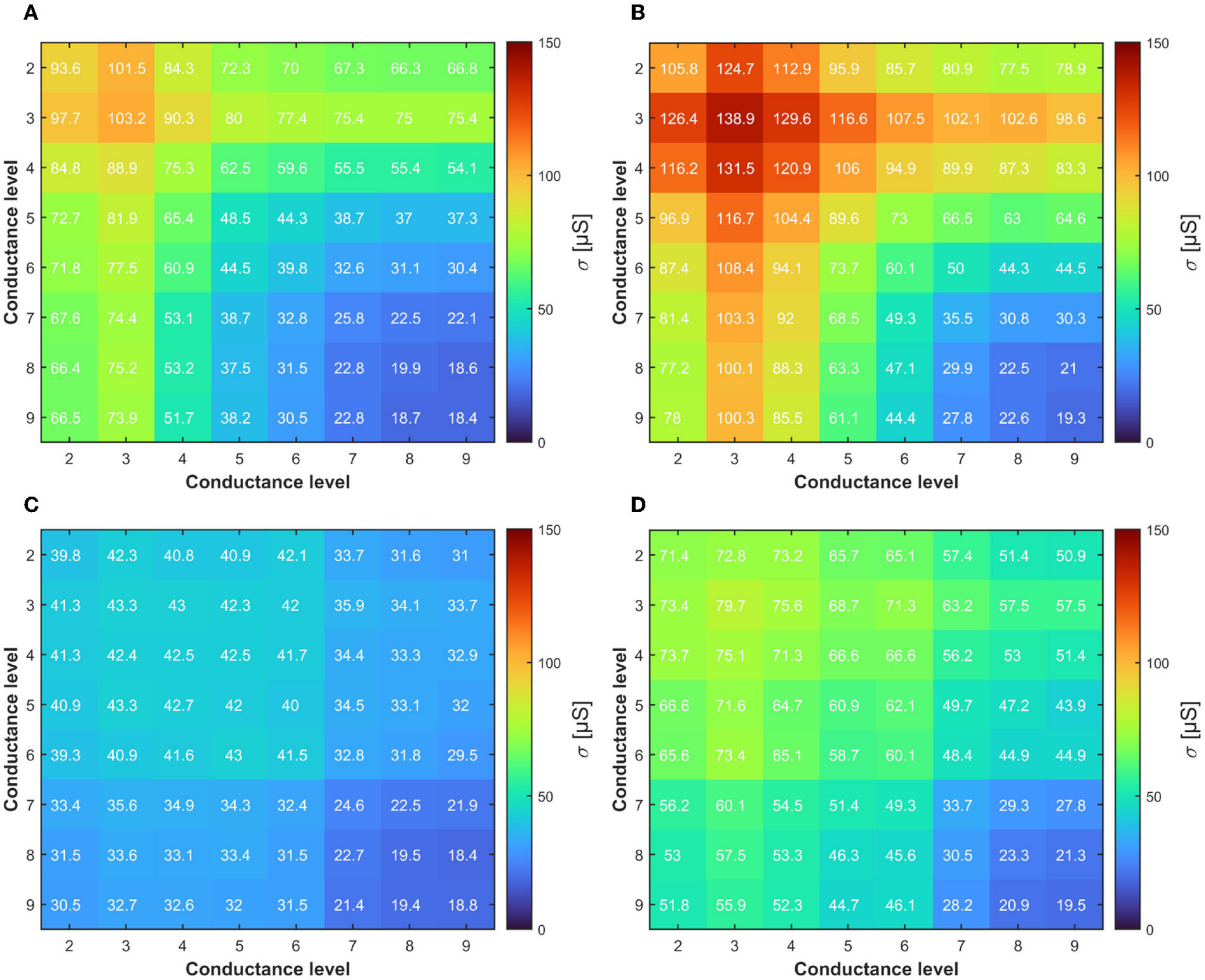
**(A)** Colormap of the conductance σ of the differential distribution obtained through ML-Set at the end of the programming algorithm. **(B)** and after 168 h evidencing the effect of the drift. **(C,D)** Same analysis performed for ML-Hybrid. The lower is better for DeepSurv accuracy.

The last operation for mapping the DeepSurv's weights on the RRAM concerns the definition of which differential operation is required to reach a certain level. Specifically, to represent a certain quantized weight *W* with one of the 17 levels, there are various combinations of *G*^+^ and *G*^−^ achieved from the 9 discrete conductance distributions of the RRAM devices. As an example, a weight of 125 μS can be obtained as the difference between *G*^+^ = 150 μS and *G*^−^ = 25 μS or equivalently as the difference between *G*^+^ = 200 μS and *G*^−^ = 75 μS. To better understand which conductance combinations allow maximizing the accuracy of the network using the 17 quantized levels, we run 2000 Monte Carlo simulations for all the possible *G*^+^, *G*^−^ cases and extract the σ for each experiment. Figure 6 shows the results of the simulations by considering the two different RRAM multilevel programming approaches described in Section 2.2. To enhance the significance of the results on a long-term basis, we also performed the simulations considering the conductance drift effect after 168 h. As it can be seen, both RRAM programming methods expose how the best mapping should take advantage of the higher conductance levels due to their enhanced stability over time (i.e., less affected by drift). This is manifested in a lower σ value which corresponds a higher accuracy of the DeepSurv network. However, achieving the 17 quantization levels also requires the use of the lower RRAM conductance levels. In this context, the programming algorithm presented in Baroni et al. (2021) allows achieving a more stable differential operation in both short and long periods. However, neural network accuracy and stability are not the only goals. Indeed, it should be reminded that the use of higher conductance levels inherently leads to lower energy efficiency, since the *I*_*C*_ required in programming operations and the read current from RRAM cells will lead to large power consumption. This exposes an interesting trade-off between the energy efficiency and the DeepSurv performances that requires a thorough design space exploration.

## 3. Results

### 3.1. RRAM-based architecture for DeepSurv

**Figure 7:**
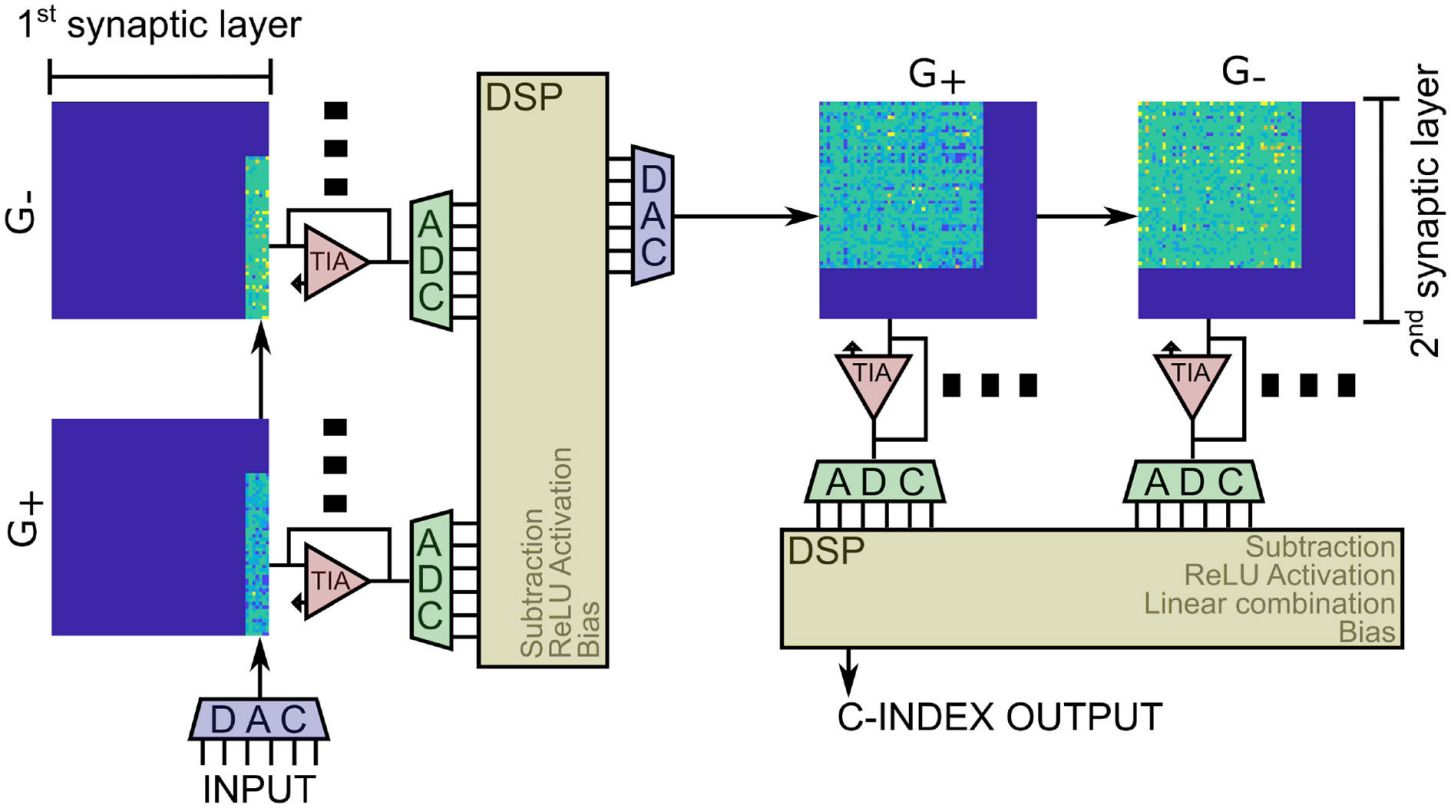
RRAM-based implementation of the DeepSurv neural network considering the 64 × 64 crossbars studied in this work. The additional circuitry, such as ADCs, DACs, and DSPs, required for the operations outside the MVMs are highlighted as well.

The implementation of the DeepSurv neural network is schematically depicted in Figure 7. It consists of a total of four RRAM arrays, two for the positive and two for the negative contribution to the synaptic weights. The input is fed into a series of DACs that control the voltage at the wordlines of the first arrays. The output of the first layer, extracted as currents, is transformed into a voltage *via* Transimpedance Amplifier (TIA), digitally converted through ADCs, and fed into a digital signal processor (DSP) that is responsible for doing the subtraction of the positive and the negative results, passing them through activation and adding the bias. The same happens for the second layer, and the second DSP is also responsible for calculating the last linear combination and exporting the C-index as the output of the computation.

From Figures 4, 6, we learned how the choice of higher conductance levels to avoid the drift in RRAM during differential operations allows achieving a more precise definition of the DeepSurv's weights. We remind that the levels from L6 to L9 are obtained with the same approach both in ML-Set and ML-Hybrid methods, whereas the L2-L5 are those to differ. In this context, ML-Hybrid demonstrates a decisive advantage in terms of conductance drifts and weight displacement countering even on a longer time span (i.e., 168 h). At the same time, we want to highlight that in all the calculations of Figure 6, L1 (obtained by reset operation) is used only in conjunction with other conductance levels to define 2 weights out of 17 and never as a starting point due to the large instability experienced in this conductance state (Puglisi et al., 2015). For this reason, on the X and Y axes of the figure, we will find only the conductive the levels from levels L2 to L9.

### 3.2. Performance and energy design space exploration for RRAM-based DeepSurv

**Figure 8:**
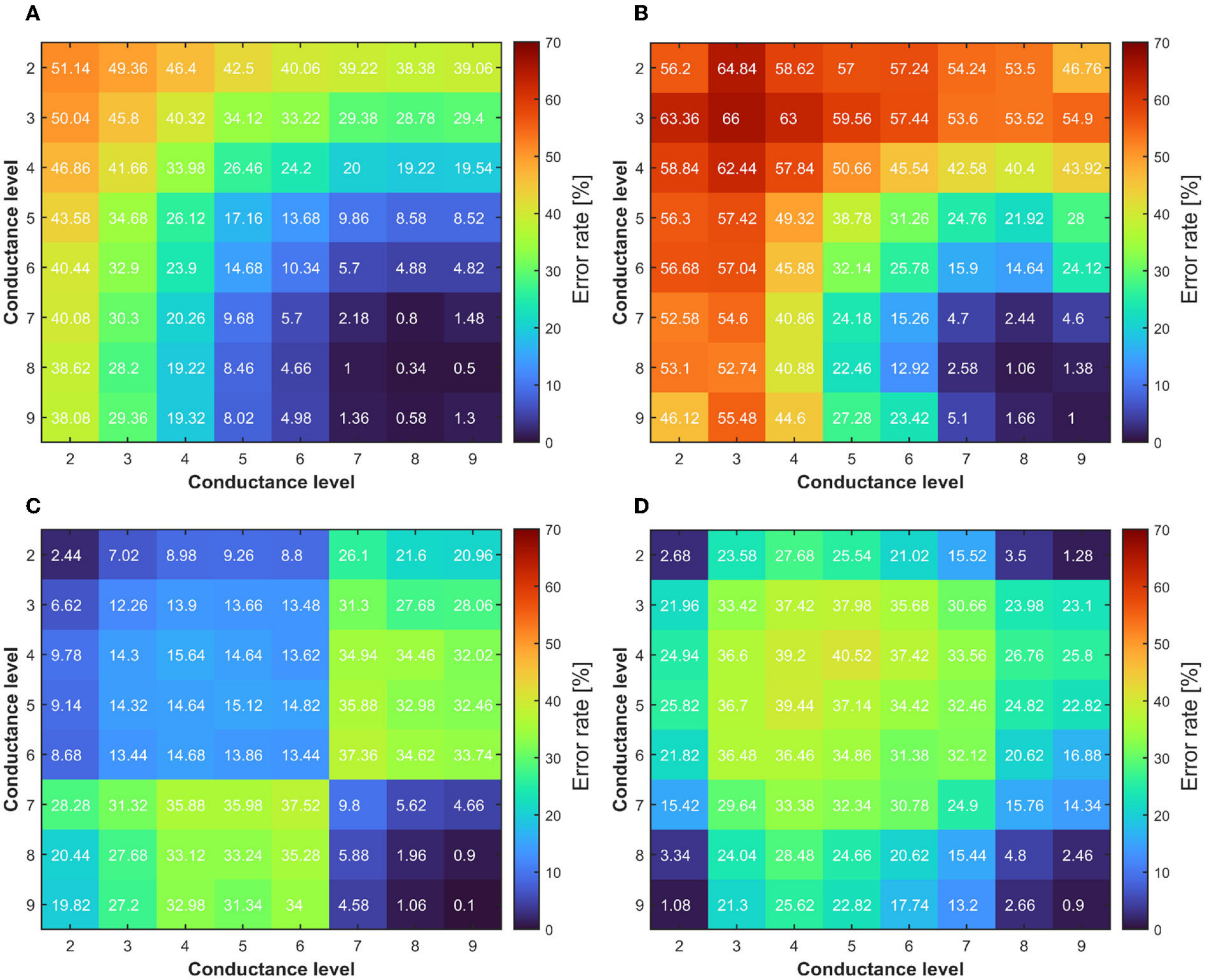
**(A)** Quantization error rate colormap of the differential distribution obtained through ML-Set at the end of the programming algorithm. **(B)** After 168 h evidencing the effect of the drift. **(C,D)** The same analysis performed for ML-Hybrid. The lower is better for DeepSurv accuracy.

**Figure 9:**
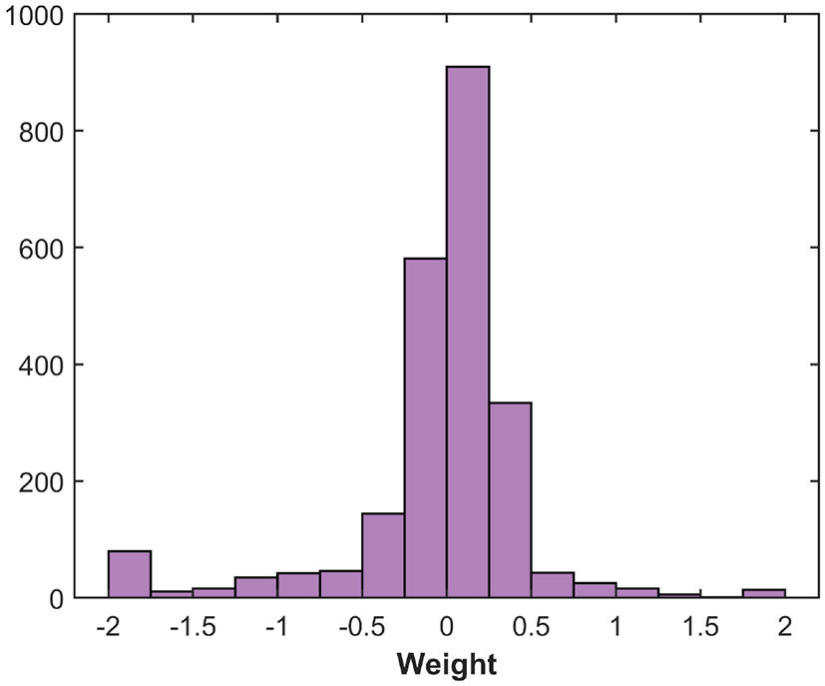
Histogram of the weight distribution.

**Figure 10:**
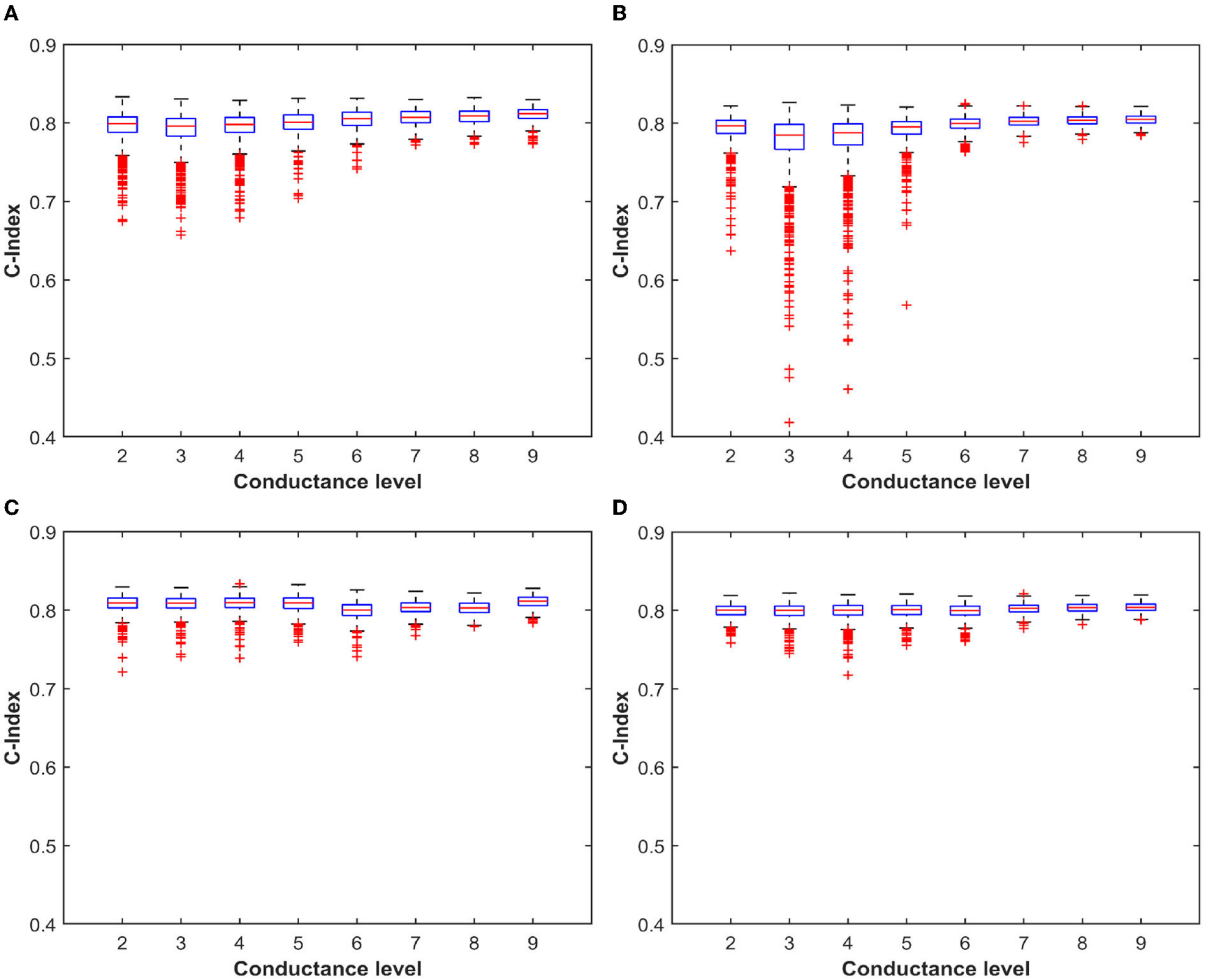
**(A)** Boxplot of the C-Index value over 1,000 simulation with the distribution obtained from ML-Set at the end of the programming algorithm. **(B)** Boxplot of the C-Index value over 1,000 simulation with the distribution obtained from ML-Set after 168 h evidencing the effect of the drift. **(C,D)** The same analysis performed for ML-Hybrid.

The multilevel programming methodologies applied to RRAM interestingly expose the neural network accuracy/energy trade-off dependently on what the weights quantization strategy targets rewarding. As an example, if the energy efficiency of the RRAM implementation is sought, we may decide to exploit a low conductance level (L2) to obtain all the possible combinations for weights mapping. On the contrary, if accuracy is the only concern, we may consider L8 or L9 as starting levels for the conductance combinations. To better understand which levels are best suited for maximizing the accuracy, we decided to extract the quantization error rate resulting from the use of each possible combination of conductive levels (L1 is used only in conjunction with other conductance levels for the reasons defined previously). Since there is a step of 25 μS between each quantized weight and the adjacent ones, we considered an upper and lower bound of 12.5 μS with respect to the target weight conductance. Figure 8 shows a trend comparable to what we have already observed in Figure 6. For ML-Set, the upper conductive levels (L8 and L9) end up being a solid choice in case we want to maximize the accuracy of the network. On the contrary, for ML-Mixed, the choice may not be immediate. We remind that the quantization error rate is not the only factor affecting the overall accuracy. In fact, this also depends on the robustness of the network itself. We, therefore, decided to study network performance in more detail. To this extent, we run 1,000 neural network simulations considering each conductance level as a starting point for obtaining the weights. The goal of this analysis is to evidence whether there are criticalities in using a particular starting conductance level. The implementation of the DeepSurv was tested either with the ML-Set or the ML-Hybrid programming methods on RRAM. Figure 10 shows the distribution of the C-index obtained shortly after programming the RRAM and after 168 h to appreciate the effect of the drift. Although the median value of the C-index obtained with the ML-Set remains acceptable for all the levels, we sometimes experience values well below 0.5 for starting conductance levels lower than L5, making them unsuitable for our application. On the other hand, we can see that with the ML-Hybrid, we are able to achieve similar median C-index values for all starting conductance levels. This important consideration defines the design point for an RRAM-based DeepSurv architecture to maintain competitive performance with reduced energy consumption. In fact, the ML-Set method can extensively use the L6 level instead of L8 or L9 to obtain and accurately map all the synaptic weights while guaranteeing a power consumption almost halved compared to the case in which the highest conductance states are used. It must be remembered that the network was trained iteratively to have weights in the range [-2:+2]. This allows an easy mapping of the characteristics of our device. The weights are distributed mainly around the value '0' as shown in Figure 9. To obtain the weights around the value '0', we can use differential operations between the levels adjacent to L6. These are also the ones that show a lower quantization error rate when compared with the others. Thanks to this property with the support of the C-index performance observed in Figure 10, we can confirm the goodness of the L6 level for this type of network. On the other hand, with the ML-Hybrid approach, it is possible to use the L2 level for similar accuracy and quantization error rate compared with the higher conductive levels (L9), thus obtaining an estimated 75% power consumption reduction. We must bear in mind that this would be possible at the expense of a doubled power consumption during the programming phase (post-training) of the RRAM array. This stems from the fact that the above-mentioned multi-level programming method is based on two combined Set/Reset procedures, thus requiring a larger energy consumption at the beginning of the operations. In any case, the greater power efficiency and performance demonstrated amortize the previous overhead as the number of inferences increases. Additionally, all these observations allow us stating that reprogramming of the devices every 168 h may be essential in the case of using ML-Set, given the strong conductance drift shown and the consequent drastic increase in sigma and quantization error, whereas the ML-Mixed algorithm relaxes this requirement, thanks to its good performance even after 168 h. It is also worth noticing that these observations were performed at room temperature. In Baroni et al. (2022), we studied the link between conductance drift, time, and temperature, evidencing that for reliable operation of the RRAM devices it is advisable to control the temperatures as much as possible since the higher the temperature, the higher will be the drift impact.

**Figure 11:**
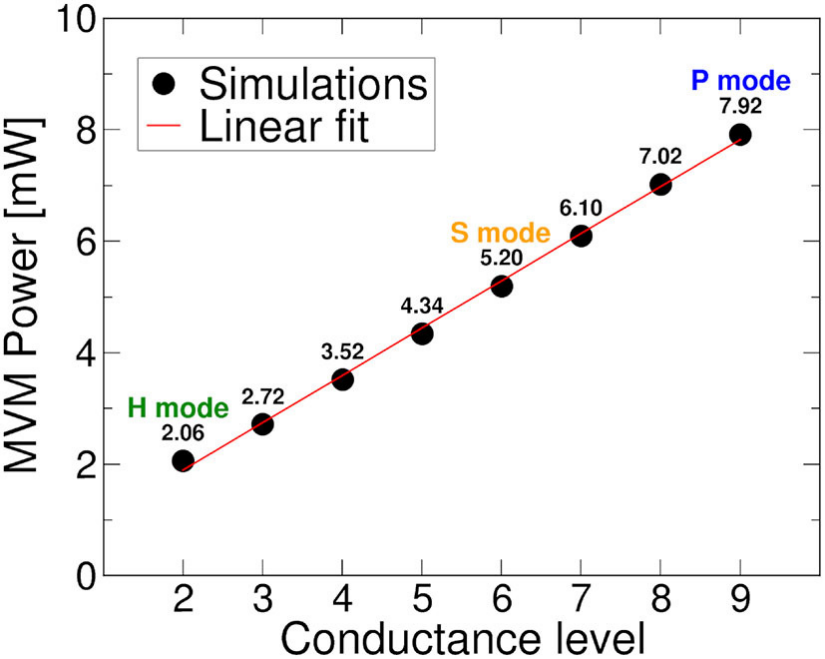
Power consumption for MVM operation as a function of the conductance level selected as starting point for differential weight computation.

Finally, we studied the power consumption drawn by the RRAM crossbar arrays involved in MVM operations during the simulation of a DeepSurv inference. Since each synaptic weight is mapped on two differential RRAM cells, we mapped the correct conductance pattern according to the chosen target programming algorithm. We defined three operation modes and considered them separately for the power analysis: *i)* a *performance* mode (P) where L9 conductance level is used to obtain the weights; *ii)* an *energy-efficient* mode (S) using ML-Set L6 conductance level; *iii)* an *energy-efficient* mode (H) using ML-Hybrid L2 conductance level. The total power drawn is computed as the sum of all the currents that flow through the crossbar arrays of the different synaptic layers, supposing to apply a readout voltage (*V*_*read*_) of amplitude that depends on the input neuron's activation value. Figure 11 shows the total power consumption for the different conductance levels used as a starting point for the differential operations, evidencing the power draw of the three presented modes.

## 4. Discussion

To compare the different IMC operation modes proposed in the previous section from the standpoints of energy consumption and efficiency, we have exploited dedicated figures of merit. We defined the throughput of the IMC system in terms of inferences per second as


(2) $$\begin{array}{r} Tr=\frac{1}{\tau_{i}} \end{array}$$

where τ_*i*_ is the latency required for the execution of an inference operation. From *Tr*, we can derive the energy consumption per inference *E*_*inf*_ as


(3) $$\begin{array}{r} E_{inf}=P_{inf}*\tau_{i} \end{array}$$

where *P*_*inf*_ is the power drawn per inference that is calculated taking into account the contributions of the crossbars used for MVM operations, the ADCs, the DACs, and the DSPs. Table 1 reports the values extracted from the literature concerning the digital circuits of the IMC architecture. The last metric adopted is the energy efficiency measured in terms of inference per watt:


(4) $$\begin{array}{r} \gamma =\frac{Tr}{P_{inf}} \end{array}$$

One of the greatest advantages of using IMC applications lies in the low power consumption that these solutions propound. In this regard, the peripheral (digital) circuitry used in this work was chosen aiming to not excessively penalize the IMC advantages in this context. The total latency of the system can be calculated through the chain of operations that the IMC architecture must perform during an inference. At the start of the chain, we have 6 DACs that take the input features of the network and convert them in parallel. Next, 2 ADCs come into play in a parallel fashion. The first converts the 48 values (48 conversion cycles) associated with the *G*^+^ matrix, the second converts the 48 values associated with *G*^−^. We assume that the ADC operation can be pipelined with the DSP. We assume that 10 clock cycles are needed by the DSP to convert the data received from the ADC. The conversion operation starts as soon as the first data from the ADC is received. This final step closes the computation steps in the first layer of our network. For the second layer, we will have an identical procedure, but here instead of the initial 6 DACs we will have to employ 48 DACs, which once again will work in parallel. By considering the latency values for the components reported in Table 1, we achieve a latency per layer of 1.48 μs. Finally, we perform the last linear combination required to provide the c-index value as output. If the DSP needs 10 clocks to execute it, we obtain a total latency value for an inference of

**Table 1:** Power consumption and latency features of the peripheral (digital) circuits considered for the simulations of the proposed IMC architecture.

**Component**	**Technology**	**Power**	**Latency**
		**consumption**	
DSP (Chen et al., 2017)	14 nm	18.35 μW	20 ns
ADC (Wang and Shi, 2018)	14 nm	41.3 μW	20 ns
DAC (Reaz and Badal, 2019)	130 nm	100 μW	500 ns


(5) $$\begin{array}{r} \tau_{i}=1,48\mu s+1,48\mu s+200ns=2.98\frac{\mu s}{inference} \end{array}$$

This holds to a *Tr* of 335,570 inferences per second.

**Figure 12:**
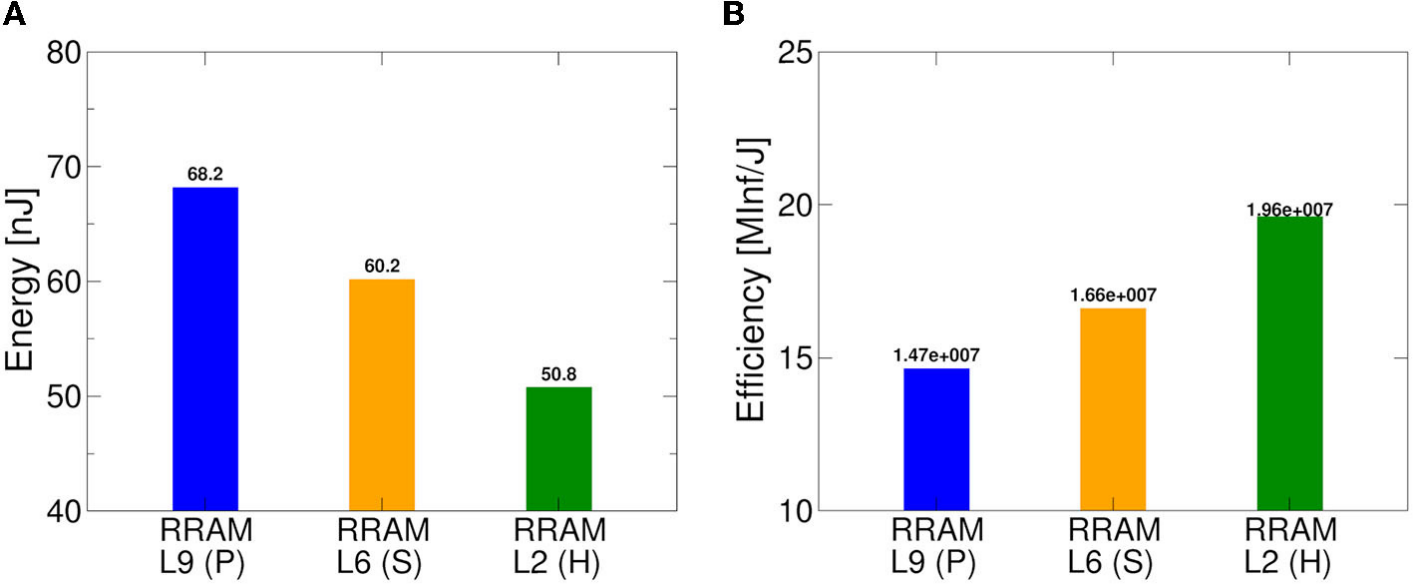
Benchmark of proposed RRAM-based IMC architecture and algorithms in terms of **(A)** energy consumption for each inference and **(B)** energy efficiency.

In Figure 12, we notice the comparison in terms of energy spent per inference and energy efficiency between the different IMC operation modes. With mode *P*, we have a power consumption only for MVM operations equal to 7.92 mW, with Mode *S* 5.2 mW, and with mode *H* 2.06 mW. Here, we can see how a careful choice of the conductive reference level can lead to energy savings per inference of around 8 nJ in the case of ML-Set and up to 18 nJ for ML-Hybrid, leading to significantly higher energy efficiency values compared to standard programming methods. This confirms the importance of a thorough analysis not only of the neural network but also of the IMC technology, starting from the programming algorithm ground up to the implementation strategies used in low power application to maximize the performance and the accuracy.

A discussion on the benefits of accelerating a survival analysis workload with the IMC architecture is then mandatory to understand the placement of this technology with respect to state-of-the-art platforms. The survival analysis, and in particular the DeepSurv application, is usually performed using commodity systems equipped with multicore CPUs, and in many cases, also with GPUs to take advantage of a potential acceleration especially when either the datasets or the number of variables in the model are exceedingly large (Nagpal et al., 2020; Li et al., 2021; Yang et al., 2022). In this work, we tried to understand if running the same DeepSurv application on different platforms (net of the porting steps required) could result in performance and energy efficiency differences. Our target application relies on a DeepSurv version that exploits Python programming language and deep learning frameworks, such as Tensorflow and Keras, to accelerate the execution of specific operations in neural networks. To get then an idea of the cost in terms of computation latency and energy consumed during the execution of a DeepSurv inference operation in a commodity system, we have run the same network implemented on our proposed IMC architecture in the COKA cluster installed at the University of Ferrara (Italy). We tried the GPU acceleration using an NVIDIA V100 card and profiled the execution using the NVIDIA *nvprof* profiler (Yang, 2020), running 1,000 inferences and measuring the execution time of all kernels running/accelerated on the GPU. While the inference code was running, we also monitored the execution through the *nvidia-smi dmon* command, which provides the power usage in Watts of the GPU with a sampling period of 1 s.

We observed that most of the execution of the inference does not show activity on the GPU, meaning that it is completely demanded on the CPU. Only some kernels (e.g., Bias, SGEMM, RELU, etc.) are fully executed on the GPUs, taking about 10 ms of the total 40 ms used for the inference. An average power consumption during the inference is reported as 38 W. These performance and power drawn metrics are not directly comparable with what we have reported for our proposed IMC architecture for several reasons, among them: *i)* we are measuring all the overheads involved in transferring the data to/from the host CPU and GPU and the time to launch kernels on GPU; *ii)* applications based on Tensorflow only run some kernels on GPU causing several additional transfers between host CPU and GPU (called host-to-device and device-to-host transfers); *iii)* our neural network model is small both in terms of input features and the number of layers, and to this extent, the execution on a GPU cannot be streamlined since is mainly affected by the time to move data in and out of the GPU memory; *iv)* regarding power drawn by the GPU, the output of *nvidia-smi* command report the values including also the consumption for moving data between host CPU and GPU without providing the power related to the inference time. Such indications make evident that GPUs are not able to efficiently accelerate applications like the DeepSurv characterized by low-latency and low-throughput. Provided the limited advantage of the GPU acceleration, we expanded the study by implementing the required network solely on an Intel Xeon 6242 CPU. The metrics extracted during the simulations reported an inference time of 38 ms and power consumption of 90 W. Given the poor figures of merit obtained, one could think to map the DeepSurv workload on a field programmable gate array (FPGA) accelerator connected to the CPU. Unfortunately, there are no implementations in the literature provided for this neural network used in survival analysis. However, we can compare the IMC potential in terms of GOP/s and GOP/J (calculated on the number of MVM operations performed during an inference) with those theoretically attainable (maximum) by an FPGA running a generic neural network inference task. We used values retrieved from different FPGA platforms obtained by a literature survey (Guo et al., 2019). In Table 2, we show the results of such a comparison. As it can be seen, while an FPGA can provide a larger boost in terms of GOP/s with respect to an IMC architecture, its energy efficiency in terms of GOP/J is comparable (most of the time, the energy efficiency is lower). Concerning the power consumption, IMC performs orders of magnitude better than FPGAs given the smaller size of the system to be integrated. We also must make some considerations on the application scenario. The DeepSurv network is designed to work with an amount of data provided in input that is often limited (i.e., individual patient's data) and does not require frequent inferences during the day (Singh and Mukhopadhyay, 2011). This allows us speculating that the power consumption and the energy efficiency take priority with respect to the inference speed performances. It should also be noted that both speed and energy efficiency in IMC depend heavily on the peripheral circuitry connected to the MVM, so we foresee a further improvement of those values as the CMOS technology used for ADC, DAC, and DSP goes in the direction of ultra-low power applications. This would favor the adoption of IMC architectures in energy-efficient systems where power consumption minimization is sought, potentially opening new application scenarios.

**Table 2:** Performance (GOP/s), power consumption (W), and energy efficiency (GOP/J) for different technologies candidated to accelerate the DeepSurv workload.

**Technology**	**Data format**	**GOP/s**	**Power consumption**	**GOP/J**
This work (IMC)	17 levels	1.82	17.1 mW	106
XC7Z045 (Qiu et al., 2016)	INT16	136.97	6.63 W	14.22
XC7Z020 (Venieris and Bouganis, 2016)	INT16	12.73	1.75 W	7.27
ZCU102 (Lu et al., 2017)	INT16	2940.7	23.6 W	124.6
XCVU440 (Shen et al., 2018)	INT16	785	26 W	30.2

*The data shown for the field programmable gate array (FPGA) were obtained (Qiu et al., 2016; Venieris and Bouganis, 2016; Lu et al., 2017; Shen et al., 2018) and represent the maximum theoretically attainable values when a generic neural network task is run*.

## 5. Conclusions

In this work, we explored the benefits of an IMC architecture for implementing an RRAM-based inference engine dedicated to a deep neural network for survival analysis of biomedical data (DeepSurv). Through the characterization of 4 kbits arrays, we evaluated the optimal methodologies to quantize and store the synaptic weights considering the drift phenomenon. A comparison of two MLC programming algorithms evidenced how it is possible to exercise a trade-off between network accuracy and energy consumption. By using a system-level simulator to map the proposed IMC architecture, we were able to demonstrate high throughput in terms of inferences per second and good energy efficiency (inf/J), maintaining the network accuracy and stability at a competitive level with Von Neumann architectures.

## Data availability statement

The raw data supporting the conclusions of this article will be made available by the authors, without undue reservation.

## Author contributions

AB, AG, and CZ have contributed equally to the planning, design, and implementation of the system, the extraction and the interpretation of the results, the figures realization, and the text writing. EP has supported the RRAM measurements. SS and EC have contributed to the setup and the measurements on the GPU cluster. PO, DI, and CW have supervised the planning and the design of this project. All authors contributed to the article and approved the submitted version.

## Funding

This work has been partially supported by the Federal Ministry of Education and Research of Germany under Grant 16ME0092. This project has also received funding from the ECSEL Joint Undertaking (JU) under Grant Agreement No. 101007321. JU receives support from the European Union's Horizon 2020 Research and Innovation Programme and France, Belgium, Czech Republic, Germany, Italy, Sweden, Switzerland, and Turkey. This work was also supported by the Italian Ministry of University and Research (MUR) and the European Union (EU) under the PON/REACT Project.

## Conflict of interest

The authors declare that the research was conducted in the absence of any commercial or financial relationships that could be construed as a potential conflict of interest. The handling editor FMP declared a past collaboration with the authors CZ, CW, PO, and DI.

## Publisher's note

All claims expressed in this article are solely those of the authors and do not necessarily represent those of their affiliated organizations, or those of the publisher, the editors and the reviewers. Any product that may be evaluated in this article, or claim that may be made by its manufacturer, is not guaranteed or endorsed by the publisher.
